# A hnRNP K–AR-Related Signature Reflects Progression toward Castration-Resistant Prostate Cancer

**DOI:** 10.3390/ijms19071920

**Published:** 2018-06-30

**Authors:** Matteo Capaia, Ilaria Granata, Mario Guarracino, Andrea Petretto, Elvira Inglese, Carlo Cattrini, Nicoletta Ferrari, Francesco Boccardo, Paola Barboro

**Affiliations:** 1 Academic Unit of Medical Oncology, Ospedale Policlinico San Martino-IRCCS, L.go R. Benzi 10, 16132 Genova, Italy; matteo.capaia@hsanmartino.it (M.C.); carlo.cattrini@gmail.com (C.C.); fboccardo@unige.it (F.B.); 2 Institute for High Performance Computing and Networking (ICAR), National Research Council (CNR), Via Pietro Castellino 111, 80131 Napoli, Italy; ilaria.granata@icar.cnr.it (I.G.); mario.guarracino@cnr.it (M.G.); 3 Core Facilities-Proteomics Laboratory, Giannina Gaslini Institute, L.go G. Gaslini 5, 16147 Genova, Italy; AndreaPetretto@gaslini.org (A.P.); elvira.inglese@gmail.com (E.I.); 4 Department of Internal Medicine and Medical Specialties, School of Medicine, University of Genova, L.go R. Benzi 10, 16132 Genova, Italy; 5 Molecular Oncology and Angiogenesis, Ospedale Policlinico San Martino-IRCCS, L.go R. Benzi 10, 16132 Genova, Italy; nicoletta.ferrari@hsanmartino.it

**Keywords:** castration-resistant prostate cancer, heterogeneous nuclear ribonucleoprotein K, androgen receptor, androgen deprivation therapy

## Abstract

The major challenge in castration-resistant prostate cancer (CRPC) remains the ability to predict the clinical responses to improve patient selection for appropriate treatments. The finding that androgen deprivation therapy (ADT) induces alterations in the androgen receptor (AR) transcriptional program by AR coregulators activity in a context-dependent manner, offers the opportunity for identifying signatures discriminating different clinical states of prostate cancer (PCa) progression. Gel electrophoretic analyses combined with western blot showed that, in androgen-dependent PCa and CRPC in vitro models, the subcellular distribution of spliced and serine-phosphorylated heterogeneous nuclear ribonucleoprotein K (hnRNP K) isoforms can be associated with different AR activities. Using mass spectrometry and bioinformatic analyses, we showed that the protein sets of androgen-dependent (LNCaP) and ADT-resistant cell lines (PDB and MDB) co-immunoprecipitated with hnRNP K varied depending on the cell type, unravelling a dynamic relationship between hnRNP K and AR during PCa progression to CRPC. By comparing the interactome of LNCaP, PDB, and MDB cell lines, we identified 51 proteins differentially interacting with hnRNP K, among which KLK3, SORD, SPON2, IMPDH2, ACTN4, ATP1B1, HSPB1, and KHDRBS1 were associated with AR and differentially expressed in normal and tumor human prostate tissues. This hnRNP K–AR-related signature, associated with androgen sensitivity and PCa progression, may help clinicians to better manage patients with CRPC.

## 1. Introduction

In the last years, experimental evidence has supported the role of the androgen receptor (AR) in castration-resistant prostate cancer (CRPC) development. Almost all patients with metastatic prostate cancer (PCa) initially treated with androgen deprivation therapy (ADT) progress to CRPC, in many cases following the reactivation of the AR pathway. Several mechanisms, which are not necessarily mutually exclusive, have been proposed to explain CRPC development [1]. They include AR amplification or overexpression, AR mutations that can modify the ligand specificity, AR gain of function, changes in the expression levels of AR coregulators, and involvement of alternative pathways that could be completely independent of AR signaling [2,3]. However, to date, the molecular mechanisms through which hormone-sensitive PCa cells acquire the ability to resist to hormone deprivation need further investigation.

Multi-omics studies showed that CRPC is a heterogeneous group of diseases characterized by different genotypes and phenotypes [4,5,6]. Emerging evidence suggests that phenotypic plasticity, driving the adaptation to ADT stress, further amplifies cellular heterogeneity and can contribute to ADT resistance [7,8]. These findings indicate that an adequate response to cytotoxic or targeted therapies cannot disregard the broad spectrum of cellular sub-clones with different clinical behaviors. Since multiple pathways are involved in CRPC development and progression, it is evident that therapies, to be useful, should selectively target driving molecular alterations at a specific stage of PCa evolution. For this purpose, we explored the possibility of developing a signature for identifying PCa and CRPC subtypes with different androgen responsiveness.

A new scenario to develop alternative targeted therapies was recently proposed by Liu et al. [9], reporting the context dependency by which AR coregulators control selectively an AR target gene set reflecting PCa biology and evolution. This finding also provides a proof of principle for the identification of PCa subtypes associated with an AR coregulator and the corresponding subset of AR-related genes.

The heterogeneous nuclear ribonucleoprotein K (hnRNP K) is a multifunctional protein playing a pivotal role in regulating numerous cellular functions, such as transcription, signal transduction, alternative splicing, and chromatin remodeling [10]. This functional versatility depends on post-translation modifications (PTM) that modulate its interactions with nucleic acids and proteins [11]. Increasing evidence for the involvement of hnRNP K in cancer progression was reported [12]. In PCa patients, we have demonstrated that its overexpression positively correlates with Gleason score and poor patients prognosis [13] and that the concomitant expression of both AR and cytoplasmic hnRNP K has a potential prognostic value [14]. In PCa cell lines, hnRNP K regulates AR activity by inhibiting its translation [15], and, in nucleoplasm, hnRNP K phosphorylation shapes the AR–DNA complex after anti-androgen treatments [16]. HnRNP K appears to be also able to regulate neuroendocrine differentiation [17].

Kelly et al. [18] underlined the importance of the adaptive phenotype acquired during ADT leading to cellular reprogramming that ultimately resulted in tumor heterogeneity and different AR status. To reproduce this behavior, we obtained the androgen-resistant cell lines PDB and MDB by treating LNCaP cell line for over one year with the anti-androgen bicalutamide (BIC) in the presence or absence of 5-α-dihydrotestosterone (DHT), respectively. Transcriptomic and proteomic analyses highlighted the high degree of phenotypic plasticity that characterizes our CRPC models and allows their adaptation under stress conditions. BIC-resistant cell lines represent two sub-populations with AR levels and subcellular localization similar to the parental LNCaP cell line, but with reduced functionality depending on AR phosphorylation status. Interestingly, partial (PDB) or minimal (MDB) AR transcriptional activity correlated with enhanced tumorigenicity and decreased sensitivity to treatment with a novel anti-androgen, enzalutamide, compared to the parental cell line [19].

Given the above findings, in this study, we investigated the role of hnRNP K in androgen-resistance using in vitro models of androgen-dependent or castration-resistant PCa. We hypothesized that hnRNP K, working like an AR transcriptional collaborator, could participate in regulating the different AR transcriptional programs during PCa development and progression. Consequently, a hnRNP K signature associated to AR activity could be useful to identify clinically distinct PCa subgroups.

## 2. Results

### 2.1. Role of hnRNP K in ADT Resistance

We previously reported that in the androgen-dependent cell line LNCaP, changes in the AR binding property of hnRNP K was associated with cell growth and AR activity [16,20]. Here, using our in vitro resistant models, we investigated the role of hnRNP K in the resistant cell lines PDB and MDB which can be considered models mimicking two CRPC subpopulations [19].

**Figure 1:**
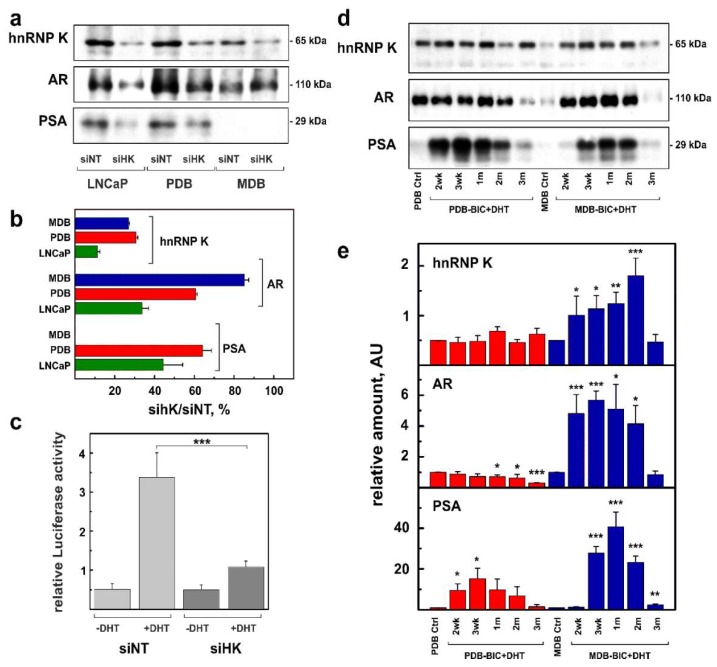
Effects of heterogeneous nuclear ribonucleoprotein K (hnRNP K) on the regulation of androgen receptor (AR) expression and AR transcriptional activity in LNCaP, PDB, and MDB cells. (**a**,**d**) Representative western blotting (WB) analysis carried out using antibodies against hnRNP K, AR, and prostate-specific antigen (PSA) and (**b**,**e**) quantitative analysis of total proteins extract from (**a**) hnRNP K-silenced (siHK) and -non-silenced (siNT) LNCaP, PDB, and MDB cell lines or (**d**) from control (Ctrl) PDB and MDB cell lines grown in the appropriate medium (see Material and Methods) or in restored LNCaP growth medium without bicalutamide (-BIC) and with 5-α-dihydrotestosterone(+DHT) for 2, 3 weeks (wk) or 1, 2, 3 months (m). The ordinate represents the mean ± SE (**b**) of the percentage of selected protein expression in hnRNP K-silenced cell lines or (**e**) the relative amounts of proteins determined by quantitative analysis. (**c**) AR transcriptional activity determined in hnRNP K-silenced and non-silenced LNCaP by the luciferase activity assay; * *p* < 0.05, ** *p* < 0.01, and *** *p* < 0.001 (Student’s *t*-test).

HnRNP K silencing decreased both AR and PSA expression in LNCaP cells (Figure 1a,b), while it was less effective in BIC-resistant cells lines, in particular MDB. Furthermore, hnRNP K silencing in LNCaP induced a 68% reduction of AR activity, as evaluated by the luciferase assay (Figure 1c). Because of their reduced AR activity [19], it was not possible to determine luciferase activity in PDB and MDB cells.

BIC removal and restored availability of androgen in the culture media determined comparable features in prostate-specific antigen (PSA) expression in both our ADT-resistant cell lines, with a maximum effect after 3–4 weeks (Figure 1d,e). However, in MDB cells, the substantial increase in PSA synthesis was associated with the overexpression of both hnRNP K and AR, while, in PDB cells, it was independent of it, probably due to AR hypersensitivity developed during ADT.

Overall, these results suggest that hnRNP K in prostate cancer cell lines is likely to act as an AR transcriptional collaborator that regulates AR activity through different molecular mechanisms depending on cell differentiation, supporting the hypothesis of the involvement of hnRNP K in ADT resistance.

### 2.2. Role of hnRNP K Phosphorylation in ADT Resistance

The mechanistic role of phosphorylation in regulating hnRNP K transcriptional activity [21,22] as well as its increased expression in several neoplasms with an aggressive phenotype [12] have been described. Here, we evaluated both its expression level and phosphorylation status in LNCaP and BIC-resistant PDB and MDB cell lines.

**Figure 2:**
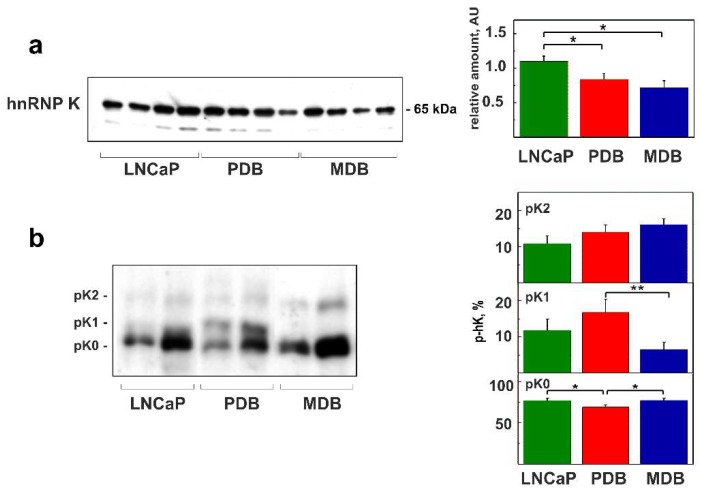
HnRNP K decreased expression and altered phosphorylation in resistant cell lines PDB and MDB compared with LNCaP. (**a**) WB analysis of hnRNP K expression in total extracts from LNCaP, PDB, and MDB cell lines. The histogram on the right side represents the mean ± SE of relative amount of proteins determined in six WBs. (**b**) The quantitative analysis of hnRNP K phosphorylation was carried out using 1D Phos-tag and WB analysis of LNCaP, PDB, and MDB total extracts. The histograms on the right side represent the mean percentage ± SE of phosphorylated hnRNP K (p-hK) evaluated in four experiments. The hnRNP K isoforms with minimal (phK0), intermediate (phK1), or maximal (phK2) phosphorylation are indicated; * *p* < 0.05 and ** *p* < 0.01 (Student’s *t*-test).

Unexpectedly, using quantitative western blotting (WB) analysis (Figure 2a), we detected a significant hnRNP K decrease in PBD and MDB cells compared to the parental cell line LNCaP, suggesting its distinct functional role in androgen-resistance compared to PCa where hnRNP K overexpression has shown diagnostic and prognostic value [13].

By evaluating the total hnRNP K phosphorylation status by means of the monodimensional phosphate affinity gel electrophoresis (1D Phos-tag), it was possible to identify a non-phosphorylated isoform (phK0) and two species characterized by intermediate (phK1) and maximal (phK2) phosphorylation (Figure 2b). As shown in the histogram of Figure 2b (right panel), significant differences were found only for PDB, showing phK0 decrease, compared to LNCaP and MDB, and phK1 increase with respect to MDB.

**Figure 3:**
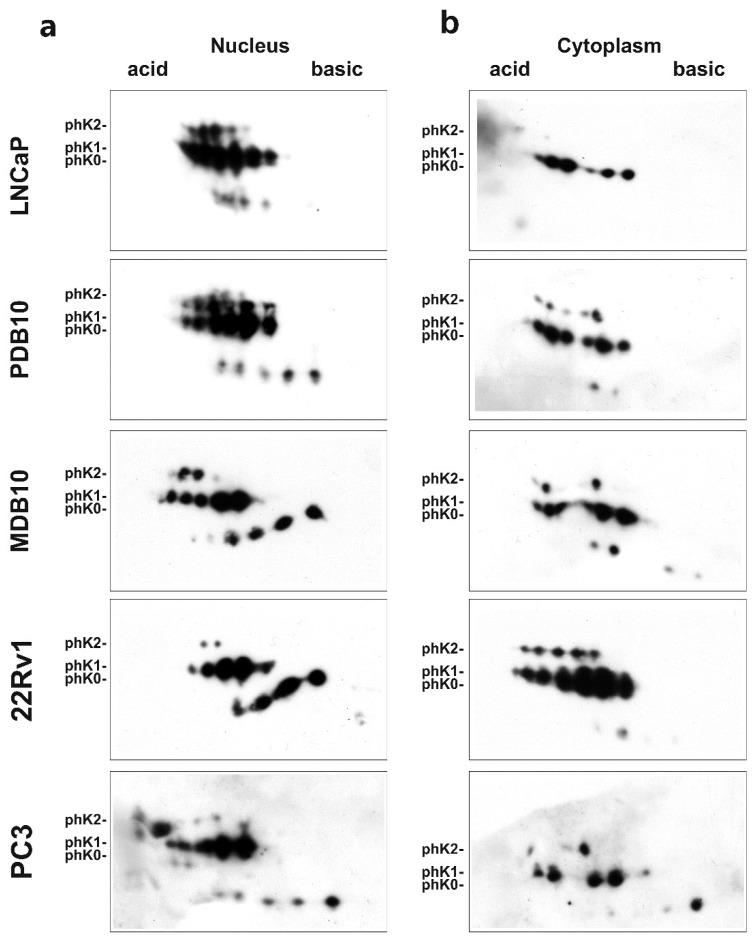
The hnRNP K phosphorylation status correlates with AR activity in prostate cancer cell lines. Nuclear (**a**) and cytoplasmic (**b**) profiles of hnRNP K phosphorylated isoforms in androgen-responsive LNCaP and resistant cell lines PDB, MDB, 22Rv1, and PC3, evaluated using 2D Phos-tag and WB analysis. The membranes were probed with an anti-hnRNP K antibody. The phK0, phK1, and phK2 identified in Phos-tag 1D (Figure 2b) are indicated.

Phosphorylation and cellular compartmentalization of hnRNP K isoforms generate a regulatory system involved in cell growth [23] and translation regulation [24]. Aberrant hnRNP K hyperphosphorylation and cytoplasmic accumulation are peculiar features of several human tumors, often associated with a worse prognosis [12]. As serine residues phosphorylation and hnRNP K functions involved in regulating its intracellular distribution, cell growth, and transcription (PhosphoSitePlus database: www.phosphosite.org) are closely related, we evaluated the role of the subcellular distribution of hnRNP K phosphorylated isoforms in PCa evolution. Using Phos-tag bidimensional gel electrophoresis (2D Phos-tag) and WB (Figure 3), we analyzed nuclear and cytoplasmic extracts from the androgen-dependent PCa cell line LNCaP and from cell lines with different CRPC phenotypes with respect to AR status and transcriptional activity: three AR-positive cell lines (PDB, hypersensitive; MDB, inactive; 22Rv1, ARv7, androgen-independent) and the AR-negative PC3 cell line [25]. Each spot detected with the anti-hnRNP K antibody was attributed, according to Kimura el al. [26], to the alternatively spliced isoforms 1 and 2 and to the phosphorylated isoforms at Ser116, Ser284, Ser353 residues (pS116, pS284, pS353), as schematically represented in Figure S1. Quantitative analysis of each spot was carried out using PDQuest software and is reported in Table 1 and Figure S2.

The level of nuclear hnRNP K phosphorylated isoform 1 was higher than that of isoform 2 in resistant cell lines compared to androgen-dependent LNCaP, while, in the cytoplasm, alternatively spliced isoforms phosphorylation was regulated in an opposite manner. Interestingly, pS353, localized in the nuclear shuttling (KNS) domains regulating hnRNP K intracellular localization, was detected exclusively in isoform 2 (Figure S2).

Quantitative analysis of nuclear hnRNP K phosphorylated forms in all cell lines revealed that: (i) pS353 decreased in resistant cell lines compared to LNCaP; (ii) an over 36% pS284 increase was observed in cell lines with active AR axis, while pS284 decreased to about 23% in MDB and PC3 cells; (iii) both high percentages of non-phosphorylated forms and low pS116 percentages were detected in androgen-independent cell lines MDB (AR-positive), 22Rv1 (ARv7), and PC3 (AR-negative), independently of the AR status.

The cytoplasmic hnRNP K isoforms distribution in the different cell lines showed that pS116 could discriminate between LNCaP and CRPC lines, regardless of AR functionality. Cytoplasmic pS353 showed an opposite trend in PDB and 22Rv1 cell lines with aberrant AR activation, in comparison to LNCaP. The pS284 isoforms were overrepresented in androgen-independent MDB and 22Rv1 cell lines.

These findings suggest that differential phosphorylation at specific serine residues and compartmentalization of hnRNP K splicing isoforms 1 and 2 correlate with different resistant phenotypes, providing further evidence for hnRNP K involvement in CRPC evolution.

### 2.3. Characterization of the hnRNP K Interactome in LNCaP, PDB, and MDB Cell Lines

HnRNP K may regulate several cellular functions, such as transcription and signal transduction, by PTMs that modify its binding partners [11,27]. Using a co-immunoprecipitation assay coupled with mass spectrometry (MS), we identified the proteins directly or indirectly interacting with hnRNP K in LNCaP, PDB, and MDB total extracts. A total of 254 proteins were identified (Table S1).

**Figure 4:**
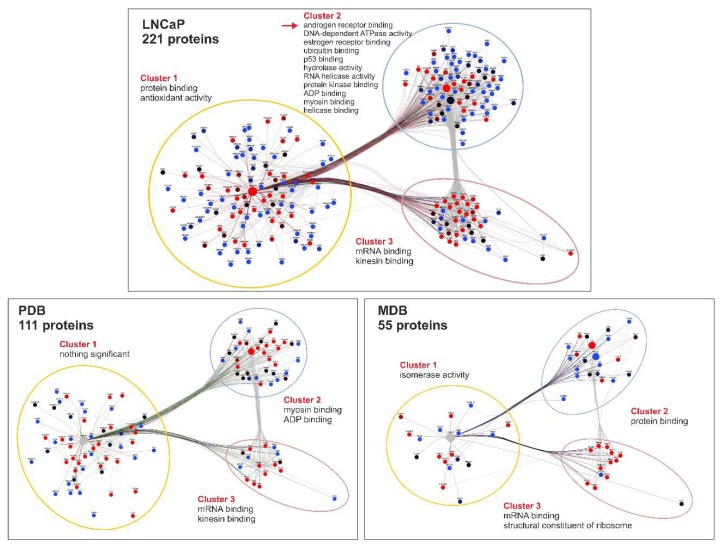
The hnRNP K interactome depends on the cellular phenotype. Protein–protein interaction networks of co-immunoprecipitates (co-IPs) obtained from LNCaP, PDB, and MDB, highlighting GO molecular function annotation restricted to prostate tissue grouped in three clusters, were realized in Cytoscape environment. The dot color refers to the Z score of protein intensity calculated on the basis of all three experiments: upregulated (**red**), downregulated (**blue**), and medium (**black**) value.

**Figure 5:**
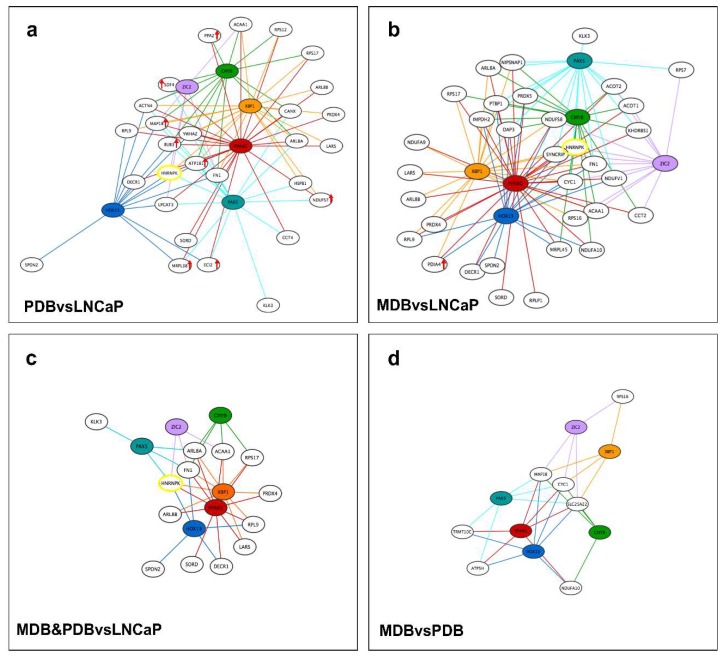
Transcriptional regulatory subnetworks of transcription factors (hK-TFs)potentially regulating the expression of differentially interacting hnRNP K proteins (DIhKPs) and selected DIhKPs, predicted and enriched by DAVID UCSC_TFBS option. The comparison of the subnetworks reveals dynamic remodeling of the hnRNP K interactome in LNCaP, PDB, and MDB. The six enriched hK-TFs are represented in colored circles, and DIhKPs in white circles, the edges are colored according to the hK-TFs. The subnetworks were grouped by the specific comparison of proteins abundance: PDBvsLNCaP (**a**), MDBvsLNCaP (**b**), MDB and PDBvsLNCaP (**c**), and MDBvsPDB (**d**). The red arrows indicate upregulated DIhKP expression; in all other cases, DIhKPs expression is downregulated.

As shown in Figure 4, the number of proteins interacting with hnRNP K gradually decreased from 221 in LNCaP, to 111 in PDB, and 55 in MDB cell lines. Protein–protein interaction networks for LNCaP, PDB, and MDB cell lines were created through Intact and Reactome databases and then clusterized through the Cytoscape app clustermaker2 [28]. Three significant clusters were obtained for each cell line, and the proteins belonging to each of them were enriched through JEPETTO Cytoscape plug-in [29], according to the Gene Ontology (GO) Molecular Function annotation restricted to prostate tissue. In cluster 1, hnRNP K was the primary hub connecting about 50% of proteins both in LNCaP and in PDB cells, while cluster 2 was predominant in the resistant cell line MDB. Interestingly, some proteins involved in AR binding (Figure 5, red arrow) were significantly clustered only in the androgen-dependent cell line LNCaP. In all cell lines, enriched GO terms in cluster 1 and 2 were different, while cluster 3, referred mainly to mRNA binding, represented about 20% of hnRNP K interacting proteins. Since, in our experiments, MS analysis was not able to efficiently detect AR, we verified hnRNP K–AR association in all cell lines using WB analysis of hnRNP K co-immunoprecipitates (co-IPs) obtained from LNCaP, PDB, and MDB (Figure S3).

By comparing hnRNP K co-IP abundance values obtained from LNCaP, PDB, and MDB, we identified three sets of differentially interacting proteins. The data were linearly modeled using Limma R package, and from the three contrasts generated (PDBvsLNCaP, MDBvsLNCaP, MDBvsPDB) we identified 51 proteins differentially interacting (*p* < 0.05) with hnRNP K (DIhKP), as reported in Table S2. Decreased interaction with hnRNP K was observed in all comparisons, except for PDBvsLNCaP, where eight proteins increased their binding capacity.

Enrichment analysis was performed to identify the biological processes and molecular functions associated to DIhKPs, according to Gene Ontology annotation (Table S3). Among the significant terms, the highest percentages corresponded to proteins involved in translation (17% for GO Biological Processes) and RNA binding (35% for GO Molecular Function), indicating that in PCa resistance, the central role of hnRNP K is linked to protein synthesis regulation.

In PCa and CRPC tissues, the altered profile of AR-interacting transcription factors (TFs) determines AR transcriptional program modifications [30]. By performing the enrichment of transcription factor binding sites (TFBS) through Database for Annotation Visualization and Integrated Discovery, we identified six TFs potentially regulating the expression of DIhKPs (hK-TFs) (Table S3). It is significant that five of these hK-TFs revealed a direct association with AR and PCa. ZIC2 and HOXB13 are AR-controlled genes [9]; the latter, in the absence of androgen, promotes androgen-independent growth in PCa cell line [31], while HOXA13 overexpression is significantly associated to PCa poor prognosis [32]. CMYB shares with AR common genes that correlate with advanced stages of PCa [33]. The TF XBP1, mediating cellular stress responses, is proposed as a predictor marker for PCa [34]. The bidirectional crosstalk between PPARG and AR regulates growth and development both in normal and in tumor prostate, as recently highlighted [35]. Using Cytoscape, we generated a network visualization of the predicted regulatory interaction between hK-TFs and 51 DIhKPs (Figure S4). In this network, PPARG and PAX5 were the main hK-TFs, regulating about 25% and 18% of DIhKPs, respectively. In addition, all DIhKPs and hK-TFs appeared to be potentially regulated by one or more hK-TF. Interestingly, hnRNP K expression resulted to be potentially regulated by four hK-TFs, among which PPARG, HOX13, and ZIC2 are controlled by AR.

As LNCaP, PDB and MDB cell lines can mimic distinct stages of PCa progression [19], we can infer that the Cytoscape network representation of the hK-TFs and the controlled DIhKPs grouped by comparing PDB versus LNCaP, MDB versus LNCaP, MDB and PDB versus LNCaP, and MDB versus PDB could represent proteins associated with different stages of PCa progression (Figure 5). Although PPARG represents the main hK-TF controlling DIhKPs in all networks, the global regulatory feature of hnRNP K partners is different in all comparisons. In PDB versus LNCaP, the eight proteins with increased hnRNP K interaction (Table S2) were under transcriptional control of PPARG and XBP1 (Figure 5a). Conversely, in MDB versus LNCaP, excluding PPARG, the number of DIhKPs controlled by others hK-TF was equally distributed (Figure 5b). Interestingly, despite the exiguous DIhKP number, the networks that featured the differences between androgen-dependent and -resistant cell lines or between MDB and PDB cell lines (Figure 5c,d) showed, respectively, an enhanced role of XBP1 and HOX13 in regulating hnRNP K partners transcription.

Overall, these findings indicate that, in cell lines with different androgen-resistant phenotypes, decreased hnRNP K expression and modified subcellular distribution of its isoforms were associated with dynamic remodeling of the hnRNP K interacting protein network depending on cellular differentiation, thus providing a rationale for DIhKPs analysis in human prostate cancer tissues.

### 2.4. Signature Identification for Potential PCa and CRPC Patient Stratification

From the above results, we hypothesized that some DIhKPs could differentiate distinct stages of PCa progression to CRPC, thus conferring clinical relevance to the findings obtained in our CRPC cell lines PDB and MDB. In this section, we describe the analytical strategies used to identify, among the 51 DIhKPs, a protein set that might putatively discriminate among clinical stages of PCa progression.

To exclude any potential bias due to the different expression of DIhKPs in the three cell lines, we verified the differential abundance calculated in our previous work [19] using total LNCaP, PDB, and MDB extracts by MS. For all proteins, the concordance between differential expression and altered hnRNP K binding capacity (respectively, differentially expressed protein—DEP—and DIhKP columns in Table S4) was evaluated, excluding 16 proteins (highlighted in light grey in Table S4) from further analysis. Moreover, referring to the expression determined by immunohistochemistry (IHC) reported in “The Human Protein Atlas” (http://www. proteinatlas.org/), we verified that only three proteins (highlighted in heavy grey in Table S4) out of the 51 DIhKPs were not expressed in normal and tumor prostate tissues. According to these findings, 32 DIhKPs were retained and considered for further evaluations.

By analyzing AR-related profiles reported in the literature evaluating AR signature [36], androgen-responsive genes [37], AR-controlled genes, and co-regulators [9,38], we verified that six out of the 32 DIhKPs retained for our analysis correlated with AR activity (Table S5).

Next, we evaluated differential expression, both at protein and transcript level, in human prostate tissues for the selected 32 DIhKPs (Table S5). Using gene expression data from four published datasets (expression profiling by array) available on Gene Expression Omnibus (GEO) portal (GSE32269, GSE101607, GSE29650, GSE66532), we performed the differential expression analyses to identify transcript alterations in tissues at different stages of PCa development. Interestingly, 53% of the 32 DIhKPs selected in our in vitro models were differentially expressed also in the first two datasets comparing non-tumoral tissue, PCa, and two subgroups of CRPC (AR-driven and non-AR-driven) bone metastases. By analyzing recent and exhaustive literature of proteomic studies in PCa tissues reported by Larkin et al. [39], we observed different expression of seven DIhKPs in PCa compared with non-tumoral or hyperplastic prostate tissues.

Combining all data reported in Table S5, we defined a hnRNP K-AR-related signature comprising eight DIhKPs selected on the basis of their AR association as coregulators and/or interactors and their differential expression in both mCRPC subgroups and PCa (Table 2).

For all proteins, evidence in the literature, reported in Table 2, shows a clear association with PCa progression. KLK3, SORD, and SPON2 expression differentiates hormone-dependent PCa from the two mCRPC subgroups. IMPDH2 tissue expression can differentiate PCa and mCRPC non-AR driven subgroups. Although there was no evidence for differential expression in androgen-dependent PCa and mCRPC tissues, the other four proteins (ACTN4, ATP1B1, HSPB1, KHDRBS1) were associated with AR. Of note, three AR co-regulators showed decreased hnRNP K interaction with respect to the parental cell line, depending on the cellular context (ACTN4 and HSPB1 for PDB and KHDRBS1 for MDB cell line). All the hK-TFs are potentially involved in regulating the transcription of the eight selected proteins. Exclusively the expression of ACTN4, an AR coregulator, is regulated by all hK-TFs, while the other proteins are controlled by one or more hK-TF.

Globally, these observations suggest that the hnRNP K–AR-related signature identified in our in vitro models might be associated to advanced stages of PCa and could identify molecular mCRPC subgroups.

## 3. Discussion

In the last years, experimental evidence has demonstrated that hnRNP K overexpression and aberrant cytoplasmic localization have an important role in PCa progression [12]. Recent findings suggest a relationship between AR and hnRNP K, as the latter regulates AR expression [57,58] and mRNA translation [15,24] and shapes a complex with AR and DNA modulated by anti-androgens [20]. In PCa tissues, the association of AR with cytoplasmic hnRNP K has a potential prognostic value [14], while the deregulation of the AKT–hnRNPK–AR–β-catenin pathway is involved in neuroendocrine differentiation [17]. Despite these evidence, the role of hnRNP K in androgen resistance has not been established yet.

HnRNP K is a modular protein that regulates a wide range of biological processes, among which transcription, splicing, and translation involved in the regulation of gene expression. By its ability to interact with both nucleic acids and proteins, hnRNP K acts like a docking platform coordinating the cross-talk between cell signaling pathways [10,21]. Consequently, the hnRNP K interactome is dynamic and changes depending on cellular compartments and external stimuli [27]. Moreover, hnRNP K functional versatility is regulated by several PTMs that modify its binding with nucleic acid and proteins [11,26].

On the basis of these premises, we explored the role of hnRNP K in androgen-resistance acquisition using three PCa cell lines: the androgen-responsive LNCaP and two resistant cell lines representing in vitro CRPC models, namely, the androgen-hypersensitive PDB and the androgen-insensitive MDB cell lines [19].

In LNCaP cell line, hnRNP K was found to regulate both AR expression and AR transcriptional activity (Figure 1a,b), while these functions were compromised in resistant cell lines, maximally in the most aggressive MDB. The adaptive phenotype characterizing PDB and MDB cells [19] under less stressful conditions (BIC removal) was found to drive a temporary recovery of AR activity which appeared to depend on the cellular context (Figure 1d,e). In the AR-inactive MDB cell line, restored androgen levels temporary reactivated the AR axis, determining a PSA rise correlated with AR and hnRNP K overexpression. Conversely, in PDB cell line, BIC removal increased PSA expression independently of AR and hnRNP K but associated with increased hnRNP K phosphorylation (Figure 2b) that could drive the AR hypersensitivity acquired during prolonged BIC exposure in androgen-containing medium.

The functional flexibility of hnRNP K arises, as for all the others hnRNPs, from the expression of alternatively spliced isoforms, PTMs, and subcellular distribution, which modify its binding activity to nucleic acids and proteins [59]. In response to mitogenic stimuli, the phosphorylated isoforms 1 and 2 generated by hnRNP K alternative splicing were differentially distributed in the nucleus and the cytoplasm [26], whereas the isoform 2 was downregulated in the colon carcinoma cell line HCT116 after p53 activation [60]. In the present study, we demonstrate a role for hnRNP K in ADT resistance acquisition depending on its alternative splicing, phosphorylation, and subcellular localization (Table 1 and Figure S2). Regardless of the AR status, the resistant cell lines showed higher levels of nuclear isoforms 1 and cytoplasmic isoform 2 than the androgen-responsive LNCaP. A quantitative comparison of the nuclear profiles of the pS116, pS284, and pS353 isoforms in the androgen-responsive LNCaP cell lines and in the resistant cell lines with different AR status and androgen responsiveness (Figure 3 and Table 1) showed a relationship between hnRNP K phosphorylation and AR transcriptional activity. In particular, to an active AR corresponded a low percentage of non-phosphorylated hnRNP K nuclear isoforms and vice versa (24% in LNCaP versus 66% in PC3). Interestingly, Ser116-phosphorylated isoforms could discriminate between resistant cell lines with different AR activity (nuclear fraction) and between LNCaP and all resistant cell lines (cytoplasmic fraction). It has been shown that the nuclear–cytoplasmic trafficking of hnRNP K may depend on Ser284 and Ser353 phosphorylation, determining inhibition of translation [24], and that Ser353 was involved in hnRNP K transactivation activity [61]. In line with these observations, our analysis showed an altered subcellular distribution of pS284 and pS353 in all CRPC cell lines in comparison to LNCaP.

Overall, these results suggest that the decreased phosphorylation of hnRNP K isoform 2, harboring the pS353 residue, and the subcellular distribution of pS116 and pS284 could have a significant role in ADT resistance.

It is increasingly evident that the identification of a molecular signature for specific stages of PCa would be useful not only for the selection of an appropriate and more effective therapy, but also for understanding the molecular basis of ADT resistance with relevant therapeutic implications [62]. The complex genomic and transcriptomic landscape of PCa has been explored through molecular analyses leading to the evidence that AR is involved in the development of drug resistance [61]. The selection pressure exerted by ADT induces alterations both in AR transcriptional program and in androgen-responsive gene (ARG) expression [37], triggering different downstream signaling pathways in PCa tissues that promote resistance [30,63]. Among the mechanisms proposed for AR reactivation during ADT, coregulators can play a critical role in influencing AR transcriptional activity and can represent new potential therapeutic targets for CRPC [64,65,66,67]. Recently, it was reported that AR coregulators control ARGs associated with different cellular processes in a context-dependent manner [9]. The dysregulated expression of AR coregulators in androgen-dependent PCa and in CRPC was reported to correlate with a poor prognosis and a more aggressive disease [65]. Therefore, it is reasonable to assume that the altered hnRNP K expression and function, by modifying the interaction with its partners involved in protein expression regulation (Figure 4 and Table S3), could alter the AR transcriptional program and androgen responsiveness in all stages of PCa.

In addition to AR coregulators, Obinata et al. [68] suggested a role for TFs in the control of the altered AR transcriptional program during anti-androgen treatment. The transcriptional regulatory networks of hK-TFs and DIhKPs subsets showed in Figure 5 confirm this hypothesis, since the composition of the hnRNP K interactome changed dynamically from LNCaP to the resistant cell lines PDB (AR-hypersensitive) and MDB (AR-inactive). Among the six hK-TFs, AR-controlled PPARG and HOX13 can be associated to ADT resistance. A recent study [35] has reported that PPARG controls prostate cell growth and differentiation and is involved in androgen-dependent PCa and CRPC evolution, acting as a tumor suppressor through its interplay with AR. The TF HOXA13, whose expression is associated with unfavorable survival [32], and the HOXB13, which controls PCa cell growth by inhibiting AR signaling [31], are also involved in PCa evolution.

**Figure 6:**
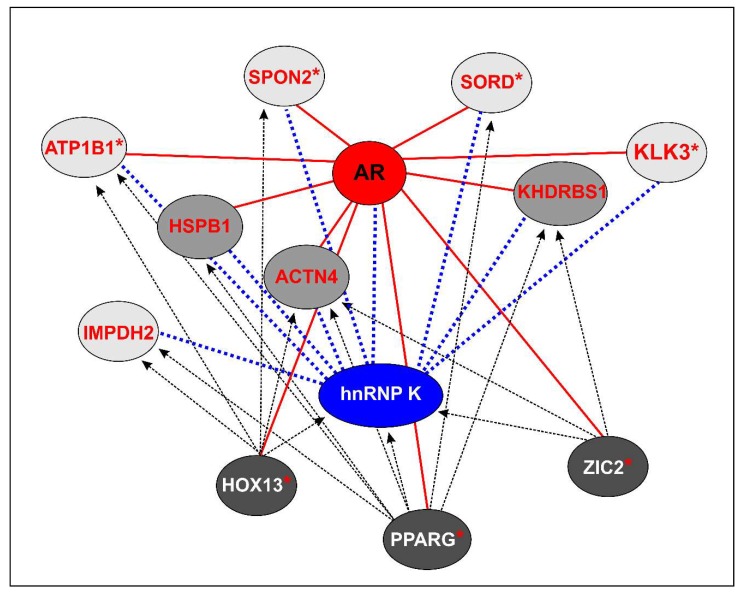
Schematic representation of the cross-talk network between hnRNP K, AR, the three AR-regulated hK-TFs, and the eight proteins selected for the hnRNP K–AR-related signature. The eight DIhKPs (heavy and light gray circles) directly or indirectly interact with hnRNP K (blue dotted lines). The hK-TFs (black circles) potentially control hnRNP K, and the eight DIhKPs (black dotted lines). AR coregulators (heavy gray circles) and androgen-responsive gene (ARGs) (highlighted with red *) are associated with AR (red solid lines).

The findings that emerge from our study demonstrate, for the first time, a clear-cut relationship between hnRNP K and AR, since, among the DIhKPs and the six hK-TFs, we found three AR coregulators (ACTN4, HSB1, KHDRBS1), four AR-controlled proteins (KLK3, SORD, SPON2, ATP1B1), and three TFs associated with AR activity (PPARG, ZIC2, HOX13) (Figure 6 and Table 2). In both androgen-resistant cell lines, the main hK-TF was PPARG which controls two distinct DIhKPs sets: in PDB (Figure 5a), the expression of eight upregulated DIhKPs was associated with the downregulation of the AR coregulators ACTN4 and HSB1, while, in MDB cell line (Figure 5b), all proteins under PPARG control were downregulated, except for PDIA4, including the AR coregulator KHDRBS1. Thus, we can infer that hnRNP K acts as a coordinator between AR and PPARG pathways and, depending on the AR coregulator involved, differently regulates the AR axis in androgen-dependent or -independent cell lines. As a confirmation, Moss et al. [69] reported different AR activity regulation by the PPARG ligand ciglitazone depending on androgen sensitivity, while Yang et al. [70] described that hnRNP K and KHDRBS1 interaction can regulate their activities in signal transduction pathways.

Understanding the context and the functional role of key proteins in ADT resistance could allow the identification of a molecular signature, rather than single proteins, that could overcome the main limit of prostate cancer treatments: tumor heterogeneity. Previously, we demonstrated that, in our CRPC model, the AR transcriptional program was modified by ADT [19]. In this study, we found that hnRNP K expression, alternatively spliced isoforms localization, and impaired phosphorylation promote ADT resistance depending on the cellular context and external stimuli. Using different analytical strategies, we identified eight proteins with dissimilar roles in the AR axis and correlation with clinical PCa progression. Of note, KLK3, SORD, and SPON2 can discriminate between non-AR-driven and AR-driven mCRPC and are characterized by common features: (i) decreased interaction with hnRNP K; (ii) control by AR; (iii) association with a single hK-TF (PAX5, PPARG, HOX13).

The eight proteins highlighted in this study (KLK3, SORD, SPON2, IMPDH2, ACTN4, ATP1B1, HSPB1, and KHDRBS1) represent a signature associated with AR modulation during the transition from androgen-dependent to castration-resistant prostate cancers. These proteins might discriminate specific stages of PCa and thus predict patients’ prognosis and response to treatments. Further investigations on PCa tissues or liquid biopsies from CRPC patients are needed to validate the clinical usefulness of the hnRNP K–AR-related signature and to evaluate whether some of the proteins included in our signature might become potential targets for new drugs development.

## 4. Materials and Methods

### 4.1. Cell Culture

The human prostate cancer cell lines LNCaP, 22Rv1, and PC3 were purchased from the American Type Culture Collection (CRL-1740, CRL-2505, CRL1435) and cultured in RPMI (Celbio, Milan, Italy) medium containing 10% fetal bovine serum, 2 mM glutamine, 1% penicillin, and 1% streptomycin. The LNCaP and 22Rv1 medium was also supplemented with 10 mM HEPES, 10 mM sodium pyruvate, and 0.25% glucose. The passage numbers at which LNCaP cells were used ranged from 22 to 30. The BIC-resistant cell lines PDB and MDB, obtained in our laboratory [19], were cultured in RPMI medium without phenol red, containing 10% charcoal-stripped fetal bovine serum, 2 mM glutamine, 10 mM HEPES, 10 mM sodium pyruvate, 0.25% glucose, 1% penicillin, 1% streptomycin, and 10 μM BIC. The PDB cell line was supplemented with 0.1 nM DHT.

### 4.2. Cell Fractionation

All buffers were supplemented with a protease inhibitors cocktail (5 mM Na_2_S_2_O_5_, 1 mM phenylmethylsulfonyl fluoride, 0.5 mM benzamidine, 20 mg/mL leupeptin, 10 mg/mL pepstatin A, 25 mg/mL aprotinin), 1 mM Na_3_VO_4_, and 1 mM dithiothreitol. The fractionation procedures were performed at 4 °C. The cells were mechanically harvested with a sterile cell scraper and washed three times in 30 mL of PBS. Total protein extracts, obtained using RIPA buffer, and cytoplasmic and nuclear extracts were prepared as reported [16,20]. After cell fractionation, the pellet was delipidated, cleaned, and solubilized for 1D or 2D gel electrophoresis as previously described [20]. The protein concentrations were determined using the Bio-Rad (Hercules, CA, USA) protein microassay with bovine serum albumin as a standard.

### 4.3. 1D and 2D Gel Electrophoresis and WB Analyses

Eight micrograms of proteins extracted from different cell fractions were loaded onto an 8–14% linear gradient (1D) or 7.5% (1D Phos-tag) polyacrylamide gels, containing 25 mM polyacrylamide -bound Mn^2+^-Phos-tag^TM^ ligand (Phos-tag Consortium, Hiroshima, Japan) and separated at 5 mA/gel for 16 h at a constant temperature of 12 °C. 2D Phos-tag was performed as described [20]. After electrophoresis separation, the proteins were transferred to a Hybond-P membrane (GE Healthcare, Piscataway, NJ, USA) and probed with anti-hnRNP K (Santa Cruz Biotechnology Inc., Santa Cruz, CA, USA; 1:5000), anti-AR (DAKO, Carpinteria, CA, USA; 1:500), and anti-PSA (Cell Signaling Technology, Danvers, MA, USA; 1:2000) antibodies at 4 °C overnight. HRP-conjugated secondary antibodies (Cell Signaling Technologies, Danvers, MA, USA; 1:2000) were used, and protein bands were detected by chemiluminescent HRP substrate (Immobilon Western, Millipore, Darmstadt, Germany) and Hyper film-ECL (GE-healthcare, Lafayette, CO, USA), according to the manufacturer’s instructions. As PDB and MDB cell lines showed a global downregulation of protein expression compared to LNCaP cell line [19], the conventional normalization approach was inadequate. Different results were in fact obtained using β-actin- or Sypro Ruby- (Invitrogen, Eugene, OR, USA) normalized data to evaluate the relative amounts of AR and hnRNP K in total extracts from LNCaP, PDB, and MDB (Figure S5). For 1D analysis, the relative amount of immunoreactive bands on WB films scanned with a GS-800 densitometer (BioRad, Hercules, CA, USA), was assessed using Bio-Rad Quantity One software and normalizing the optical density of each band to the total optical density of the corresponding lane in the Sypro Ruby-stained gel providing the total protein content [16]. In Figure S6, the Sypro Ruby-stained gels used for quantitative analysis of WB reported in Figure 1a,d and Figure 2a are shown. The quantitative analysis of 1D Phos-tag WB was carried out comparing the relative percentage of phK0, phK1, and phK2 hnRNP K isoforms in each cell lines. The 2D Phos-tag analyses, including detection, alignment, and matching for spots present in all 2D maps, were done using the software package PDQuest (ver. 8.6, BioRad, Hercules, CA, USA).

Statistical significance was evaluated by two-tailed Student’s *t*-test within OriginPro 7.5 software (OriginLab, Northampton, MA, USA), and the differences were considered statistically significant when *p* < 0.05.

### 4.4. hnRNP K Silencing and Reporter Assay

ON-TARGET plus SMART pool for human hnRNP K (Dharmacon, GE- healthcare, Lafayette, CO, USA) was used to knockdown the expression of hnRNP K; siCONTROL non-targeting (NT) siRNA pool (Dharmacon, Lafayette, CO, USA) was used as a negative control. The cells were grown in their proper medium and transfected with Lipofectamine 2000 (Thermo Fisher, Waltham, MA, USA) following the manufacturer’s instructions. Protein expression was analyzed by WB 72 h after transfection.

To measure AR function, the cells were transfected with Lipofectamine 2000 utilizing the Cignal androgen receptor dual-luciferase reporter kit (Qiagen, Hilden, Germany), following the manufacturer’s instructions. Luciferase activity was assayed in triplicate 48 h after transfection using the dual-luciferase reporter assay kit (Promega, Madison, WI, USA). To control for general effects on transcription, Renilla luciferase was co-transfected in all reporter assays, and luciferase values represent the ratio luciferase/Renilla.

### 4.5. Co-Immunoprecipitation

Co-immunoprecipitation, employed to identify proteins directly or indirectly interacting with hnRNP K, was carried out using the Pierce Direct IP kit according to the manufacturer’s instructions (Pierce Biotechnology, Rockford, IL, USA). Briefly, total cellular extracts, obtained with IP Lysis/Wash buffer containing a protease inhibitor cocktail, were pre-cleared with agarose resin and passed through an AminoLink plus resin with covalently immobilized anti-hnRNP K antibodies (Santa Cruz Biotechnology, Santa Cruz, CA, USA). The eluted proteins were subjected to MS for identification of the hnRNP K interactome. Control resin and quenched antibody-coupling resin without anti-hnRNP K antibodies were utilized to identify non-specific interactions. Common proteins between these groups and the co-IPs were excluded from bioinformatic analyses. For MS analysis, three independent experiments with LNCaP, PDB, and MDB were performed.

The hnRNP K co-IPs were analyzed by WB for hnRNP K and AR expression evaluation in LNCaP, PDB, and MDB cell lines (Figure S3).

### 4.6. MS Analysis

The samples were processed by the FASP Protein Digestion Kit (Expedeon, Harston, UK). Briefly, the co-IP samples were mixed with 0.3 mL of 8 M urea in 0.1 M Tris/HCl pH 8.5 (UA solution), loaded into the filtration devices, and alkylated in 0.1 mL of 50 mM iodoacetamide in UA solution for 1 h in darkness at room temperature. The samples were digested using, sequentially, 1 µg of LysC and 3 µg of Trypsin in 50 mM NaHCO_3_ solution at 37 °C overnight. Peptides were collected by centrifugation of the filter units for 10 min, and the filter devices were rinsed with two 40 µL washes of 50 mM NaHCO_3_ and 50 µL 0.5 M NaCl to eliminate the hydrophobic interactions. Each sample digest was desalted on StageTips and analyzed by liquid chromatography–tandem mass spectrometry (LC–MS/MS).

All MS experiments were performed on a nanoscale high-performance liquid chromatography system connected to a hybrid linear trap quadrupole (LTQ) Orbitrap mass spectrometer. The MS instrument was operated in data-dependent mode to automatically switch between full-scan MS and MS/MS acquisition. Survey full-scan MS spectra were acquired in the Orbitrap analyzer with resolution *R* = 60,000. The 10 most intense peptide ions with charge states ≥2 were sequentially isolated and fragmented by collision-induced dissociation in the LTQ mass spectrometer. The raw mass spectrometric data were analyzed using the MaxQuant pipeline. MaxQuant enables high peptide identification rates, individualized p.p.b.-range mass accuracies, and proteome-wide protein quantification [71]. The statistical and the pathways analyses were done with the freely available Perseus [72] and Cytoscape software [73] respectively.

### 4.7. Network Analysis

Cytoscape (v 3.6.0) was used to visualize the protein–protein interaction network obtained from the above proteomic data (co-IP) and to highlight functional annotations [28]. Statistically significant proteins were used as the queries to interrogate Intact and Reactome database in order to obtain a protein–protein interaction network. The network was treated as undirected, and all duplicated edges and self-loop were removed. To highlight the most important proteins, a network analysis was performed. In particular, the degrees and closeness were assessed. The degree is the simplest topological index, corresponding to the number of nodes adjacent to a given node, where “adjacent” means directly connected. The degree allows an immediate evaluation of the regulatory relevance of the node. The closeness is a node centrality index. The closeness of a specific node is calculated by computing the shortest path between the node and all other nodes in the graph and then calculating the summa. From the biological point of view, the closeness can be interpreted as the “probability” of a protein to be functionally relevant for several other proteins. Moreover, the MaxLFQ expression data were loaded into the network as attribute node. Subsequently, a clusterization of significant proteins related to the expression data and to the topology of the network was performed as previously described, by clustermaker2 (Cytoscape App) [28] with the “autoSOME Clustering” algorithm (node attribute = expression; distance metric: Euclidean, ignore node with no data). Each obtained cluster was functionally tagged by an enrichment, performed by JEPETTO [29] (network: PSICQUIC, reference database: GO MF/prostate tissue) if statistically significant results were obtained.

### 4.8. Differential Expression and Enrichment Analyses

Differential expression analyses were carried out both on protein detected through MS spectroscopy for their binding to hnRNP K and on gene expression data extracted from four datasets published and available on the GEO portal (GSE32269, GSE101607, GSE29650, GSE66532).

In the first case, the log-transformed values of protein binding were imported and analyzed through linear model fitting and empirical Bayes approach provided by Limma R package [74]. Three different contrast matrices were generated: PDB versus LNCaP, MDB versus LNCaP, MDB versus PDB. Proteins showing a *p*-value ≤ 0.05 and a |log_2_FC| ≥ 1 were considered differentially interacting (DIhKPs).

Microarray data from GEO datasets were imported through GEOquery R package [75], “GEOquery: a bridge between the Gene Expression Omnibus (GEO) and BioConductor”, and comparison analysis of genes was performed using Limma.

For each dataset, pairwise comparisons between sample groups, as indicated in Table S5, were performed. Genes having a |log_2_FC| ≥ 1 and the corresponding *p*-value ≤ 0.05 were considered statistically significant.

Enrichment analysis was performed on the 51 DIhKPs to identify the over-represented GO annotations regarding Biological Processes and Molecular Function, as well as the transcription factor binding sites through the category UCSC-TFBS provided by the functional annotation tool DAVID v 6.8 [76].

## Figures and Tables

**Table 1:** Quantitative analysis of the spots reported in Figure 3. The assignment of the alternatively spliced isoforms 1 and 2, non-phosphorylated, and S116-, S284-, S353-phosphorylated forms was carried out according to the schematic representation showed in Figure S1.

Cell Line	LNCaP(AR-FL ^1^)	PDB(AR-FL)	MDB(AR-FL)	22Rv1 (ARv7 ^2^)	PC3(AR-Null ^3^)
AR Status	Active	Hypersensitive	Inactive	Androgen Independent	Not Detected
**Nucleus**					
Alternatively spliced					
isoform 1 (%)	50.1	66.7	62.5	76.6	66.8
isoform 2 (%)	49.9	33.3	37.5	23.4	33.2
Phosphorylated forms					
pS116 (%)	17.1	19.5	15.0	1.7	0.7
pS284 (%)	37.6	45.8	23.2	36.1	22.8
pS353 (%)	21.6	8.4	16.2	1.8	10.6
Non-phosphorylated forms (%)	23.6	26.2	45.5	60.3	65.8
**Cytoplasm**					
Alternatively spliced					
isoform 1 (%)	38.7	45.1	66.1	63.7	76.2
isoform 2 (%)	61.3	54.9	33.9	36.3	23.8
Phosphorylated forms					
pS116 (%)	0.0	8.2	9.9	13.3	6.8
pS284 (%)	0.0	0.0	42.0	41.3	0.0
pS353 (%)	24.9	35.7	21.5	17.8	23.8
Non-phosphorylated forms (%)	75.1	56.1	26.6	27.7	69.4

^1^ Cell line expressing AR full length; ^2^ Cell line expressing AR splicing isoform ARv7; ^3^ Cell line not expressing AR.

**Table 2:** Selected DIhKPs associated with hK-TF, AR, PCa and differential expression in human prostate tissues, delineating a hnRNP K–AR-related signature.

Gene symbol/Protein Name	Differential hnRNP K Interaction	Associated hK-TF	Association with AR	Human Prostate Tissues Differential Expression				Association with PCa (Literature Evidences)
				Proteomic Studies	Transcriptomic Studies			
KLK3/PSA	MDB&PDB vs. LNCaP **↓** *	PAX5	AR controlled	PCa vs. BPH **↓**	PCa & mCRPC vs. NT **↑**	mCRPC vs. PCa **↓**	mCRPC non-AR driven vs. AR driven **↓**	Biomarker [40,41,42]
SORD/Sorbitol dehydrogenase	MDB&PDB vs. LNCaP **↓**	PPARG	AR controlled		PCa vs. NT **↑**	mCRPC vs. PCa **↓**	mCRPC non-AR driven vs. AR driven **↓**	Increased expression in high Gleason PCa; reduced expression after castration [43]
SPON2/Spondin2	MDB&PDB vs. LNCaP **↓**	HOX13	AR controlled				mCRPC non-AR driven vs. AR driven **↓**	Biomarker [44,45]
ATP1B1/Sodium-potassium-transporting ATPase subunit beta1	PDB vs. LNCaP **↑** **	PPARG, XBP1, PAX5, HOX13, CMYB	AR controlled					Frequently overexpressed and amplified in CRPC [46]
ACTN4/Actinin4	PDB vs. LNCaP **↓**	PPARG, XBP1, PAX5, HOX13, ZIC2, CMYB	AR coactivator, direct interaction		PCa vs. NT **↓**			Reduced expression in high-grade PCa nuclei [42,47,48]
HSPB1/HSP27	PDB vs. LNCaP **↓**	XBP1, PAX5	AR coactivator, direct interaction	PCa vs. NT & BPH **↑**	PCa vs. NT **↑­**			Role in PCa epithelial to mesenchymal transition and resistance [49,50,51]
KHDRBS1/Sam68	MDB vs. LNCaP **↓**	PPARG, PAX5, ZIC2, CMYB	AR coactivator/corepressor					Role in regulating AR splice variant, cell proliferation, and survival to chemotherapeutic agents [52,53,54]
IMPDH2/Inosine-5-monophosphate dehydrogenase 2	MDB vs. LNCaP **↓**	PPARG, XBP1, PAX5, HOX13, CMYB		PCa vs. NT **↑**	PCa & mCRPC vs. NT **↑**		PCa vs. mCRPC non-AR driven **↑**	Role in PCa metastasis and progression [55,56]

↓* downregulated; **↑** ** upregulated.

## Supplementary Materials

Supplementary materials can be found at http://www.mdpi.com/1422-0067/19/7/1920/s1.

## Abbreviations

ADT	Androgen deprivation therapy
AR	Androgen receptor
ARG	Androgen-responsive gene
BIC	Bicalutamide
Co-IP	Co-immunoprecipitate
CRPC	Castration-resistant prostate cancer
DAVID	Database for Annotation Visualization and Integrated Discovery
DEP	Differentially expressed protein
DIhKP	Differentially interacting hnRNP K protein
DHT	5-α-dihydrotestosterone
GO	Gene ontology
hK-TF	Transcription factor potentially regulating the expression of differentially interacting hnRNP K protein
LTQ	Linear trap quadrupole
MS	Mass spectrometry
PTM	Post-translation modifications
TF	Transcription factor
TFBS	Transcription factor binding sites
PAGE	Polyacrylamide gel electrophoresis
PCa	Prostate cancer
WB	Western blotting
